# Decoding the Uric Acid to HDL Cholesterol Ratio (UHR) as a Novel Metabolic Biomarker for Chronic Pain: Insights Into Precision Diagnostics and Management

**DOI:** 10.1155/prm/7350322

**Published:** 2026-08-18

**Authors:** Yanjun Liu, Jianwei Wang, Zifeng Xu

**Affiliations:** ^1^ Department of Anesthesiology, the International Peace Maternity and Child Health Hospital, School of Medicine, Shanghai Jiao Tong University, 910 Hengshan Road, Shanghai 200030, China, sjtu.edu.cn; ^2^ Shanghai Key Laboratory of Embryo Original Diseases, Shanghai 200030, China, sjtu.edu.cn

**Keywords:** biological biomarkers, chronic pain, cross-sectional study, inflammation, uric acid to HDL ratio (UHR)

## Abstract

**Background:**

Chronic pain (CP) constitutes a multidimensional clinical phenomenon with rising prevalence globally, producing substantial societal and healthcare burdens. Metabolic and inflammatory dysregulations are central to its pathophysiology, stimulating interest in identifying biomarkers that reflect these underlying processes. The uric acid to high‐density lipoprotein cholesterol ratio (UHR) serves as an integrated index representing systemic inflammatory status and lipid metabolism, offering potential relevance in CP research.

**Methods:**

This cross‐sectional study analyzed data from 12,012 adults aged ≥ 20 years from the National Health and Nutrition Examination Survey (NHANES) 1999–2004 cycles. UHR was calculated as serum uric acid divided by high‐density lipoprotein cholesterol. CP was defined as pain lasting ≥ 24 h during the previous month and persisting for ≥ 3 months. Associations between UHR and CP were evaluated using multivariable logistic regression models adjusting for demographic, metabolic, and clinical covariates. Restricted cubic spline and threshold‐effect analyses were further performed to assess potential nonlinear relationships.

**Results:**

Higher UHR levels were associated with increased CP prevalence. In the fully adjusted model, participants in the highest UHR quartile exhibited significantly greater odds of CP compared with those in the lowest quartile (OR = 1.44, 95% CI: 1.22–1.70). Continuous‐variable analysis demonstrated that each unit increase in UHR was associated with an 11% increase in CP odds. Restricted cubic spline analysis revealed an approximately linear positive association between UHR and CP prevalence, whereas threshold‐effect analysis did not identify a statistically significant nonlinear relationship.

**Conclusion:**

The findings indicate that UHR may serve as a biologically informative metabolic correlate of CP. While causality cannot be inferred due to the cross‐sectional design, these results highlight a link between metabolic‐inflammatory status and CP prevalence. Future longitudinal studies are required to clarify temporal dynamics and assess the predictive value of UHR.

## 1. Introduction

Chronic pain (CP) represents a widespread and debilitating health condition whose prevalence is steadily increasing worldwide, creating considerable social and economic burdens [1–3]. Defined as discomfort that persists beyond the expected timeframe for tissue healing, CP is commonly operationalized as pain lasting more than 3 months, particularly in the absence of other contributing medical conditions [4–6]. Rather than being solely a secondary manifestation of other diseases, CP is increasingly recognized as a primary healthcare concern requiring dedicated attention [7, 8]. Its impact is extensive, affecting patients’ quality of life [9], social interactions and environment [10], and healthcare infrastructures [11, 12]. Moreover, CP involves multiple dimensions, encompassing sociodemographic features [13], psychological comorbidities [14], clinical presentations [15], and biological characteristics [16–18].

Uric acid (UA), a metabolic byproduct of purine catabolism, exerts diverse physiological effects, including antioxidant and pro‐oxidant actions [19, 20], modulation of inflammatory responses [21, 22], regulation of nitric oxide availability [23, 24], interaction with immune mechanisms [25, 26], and potential involvement in the aging process [27, 28]. Under normal physiological conditions, UA functions as an antioxidant, neutralizing reactive oxygen species and safeguarding cellular integrity [29]. In contrast, high‐density lipoprotein (HDL) is recognized for its cardiovascular protective roles, particularly via reverse cholesterol transport, which facilitates cholesterol clearance from arterial walls and mitigates atherosclerosis risk [30–32]. The UA to HDL cholesterol ratio (UHR) thus provides an integrated metric reflecting systemic metabolic and inflammatory status. Previous studies have demonstrated associations between UHR and numerous conditions, including atherosclerosis [33], ischemic heart disease [34], hypertension [35], coronary artery disease [36], insulin resistance, visceral adiposity, diabetes, metabolic syndrome, and metabolic dysfunction‐associated nonalcoholic fatty liver disease [37–40].

Considering the well‐established relationship between CP and inflammation, these findings motivate an investigation into whether UHR correlates with CP. To date, however, no studies have specifically examined the link between UHR and CP. Against this context, the present study aimed to evaluate the association between UHR and CP in a nationally representative sample of US adults. We hypothesized that higher UHR, indicative of relatively elevated UA and lower HDL levels, would be associated with an increased likelihood of reporting CP. The study’s objective was to determine whether UHR serves as a biomarker linked to CP and to provide epidemiologic evidence that could inform future mechanistic and longitudinal research on this complex condition.

## 2. Materials and Methods

### 2.1. Study Design and Participants

**FIGURE 1:**
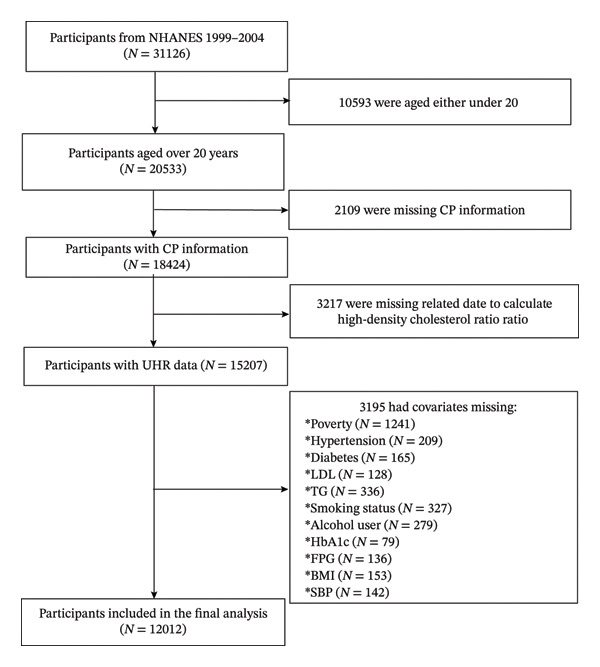
Flowchart illustrating the selection and inclusion of participants in the study.

This study utilized data from the National Health and Nutrition Examination Survey (NHANES; https://www.cdc.gov/nchs/nhanes/), a nationwide cross‐sectional study in the United States that employs a complex, multistage, stratified probability sampling design to represent the noninstitutionalized US population (Supporting Table 1). The NHANES dataset includes demographic information, anthropometric measurements, blood biochemistry, and survey‐based questionnaires. All participants provided informed consent at the time of data collection, thus obviating the need for additional ethical approval. Figure 1 illustrates the participant selection process (Figure 1). Initially, demographic, laboratory, physical examination, and survey data were collected from 31,126 participants in NHANES cycles spanning 1999–2004. Since CP questionnaires were only administered to individuals aged ≥ 20 years, 10,593 participants under 20 were excluded. Additional exclusions comprised 2109 participants without relevant CP information and 3217 participants lacking sufficient data to calculate UHR. To improve analytic accuracy, we further removed 3195 participants with missing values for potential confounders, including poverty income ratio (PIR) (*n* = 1241), hypertension (*n* = 209), diabetes status (*n* = 165), low‐density lipoprotein (LDL) (*n* = 128), triglycerides (TG) (*n* = 336), smoking history (*n* = 327), glycated hemoglobin (HbA1C) (*n* = 79), fasting plasma glucose (FPG) (*n* = 136), body mass index (BMI) (*n* = 153), systolic blood pressure (SBP) (*n* = 142), and alcohol consumption (*n* = 279). Consequently, the final analytic sample comprised 12,012 participants, derived using a complete‐case approach. Although this method ensured consistency across multivariable models, it may reduce generalizability and introduce selection bias if excluded participants differed systematically from those retained.

### 2.2. Assessment of UHR in the Study

Two biochemical measurements were extracted from the NHANES dataset for each participant: serum UA and HDL, both quantified in mg/dL. The UHR was calculated by dividing serum UA by HDL, yielding a composite marker for exploring potential associations with CP.

### 2.3. Identification of CP

CP was operationalized using NHANES pain questionnaire items. Only participants aged ≥ 20 years completed these items. Specifically, CP was defined using two questionnaire variables: MPQ100 (“Did you have any pain that lasted more than 24 h in the past month?”) and MPQ110 (“Duration of pain”). Consistent with previous epidemiologic approaches, participants reporting pain ≥ 24 h in the past month and persisting for ≥ 3 months were classified as the CP group, while those without reported pain or with pain < 3 months were classified as non‐CP. This definition approximates CP status in a large epidemiologic dataset, though it may include heterogeneous pain phenotypes. Pain site, severity, recurrence, and underlying etiology were not uniformly available, so some misclassification is possible. The operational definition aligns broadly with duration‐based epidemiologic definitions rather than formal clinical pain phenotyping.

### 2.4. Covariates

Building on previous research, this study incorporated a comprehensive set of variables to adjust for potential confounding factors, including TG, HbA1C, age, LDL, PIR, waist circumference (WC), hypertension, SBP, FPG, BMI, diabetes status, race, and gender. Covariates included in the fully adjusted model were selected a priori based on prior epidemiologic studies, biological plausibility, clinical relevance, and availability within the NHANES framework, considering factors potentially associated with both UHR and CP. Demographic characteristics were retained to account for baseline population heterogeneity, whereas adiposity‐, glycemic‐, lipid‐, and blood pressure‐related variables were incorporated to reduce confounding in the relationship between UHR and CP. Overall, covariate selection was guided by a structured epidemiologic and clinical rationale rather than automated or purely significance‐driven criteria.

Hypertension was defined as meeting any of the following conditions: a SBP of 140 mmHg or higher, a diastolic blood pressure of 90 mmHg or higher based on physical measurements, or self‐reported physician diagnosis of hypertension in the blood pressure questionnaire. Meeting any one of these criteria was sufficient to classify a participant as hypertensive. Race was categorized according to NHANES survey classifications: Mexican, Hispanic, White, Black, and Other. BMI was calculated using the standard formula, dividing weight in kilograms by height in meters squared (BMI = kg/m^2^).

Diabetes was defined by fulfilling any of the following: FPG ≥ 7 mmol/L, HbA1C ≥ 6.5%, self‐reported physician diagnosis of diabetes, or reported use of hypoglycemic medications. Meeting any one of these criteria was sufficient to define a participant as having diabetes. These definitions ensured consistency with prior epidemiologic studies and provided a standardized framework for adjusting for metabolic and demographic covariates in the analysis.

### 2.5. Missing Data Management

NHANES demographic, laboratory, examination, and questionnaire datasets from each survey cycle were merged using the unique participant identifier (SEQN) prior to pooled analyses across the 1999–2004 survey cycles. A complete‐case analysis strategy was applied. Participants with missing data for exposure variables, CP status, or covariates included in the fully adjusted models were excluded prior to statistical analyses.

### 2.6. Statistical Description

Continuous variables are presented as medians with interquartile ranges (Q1–Q3), whereas categorical variables are summarized as counts and percentages. Between‐group comparisons for continuous variables were performed using the Kruskal–Wallis test, while categorical variables were compared using the chi‐square test. All statistical tests were two‐sided, and a *p* value < 0.05 was considered statistically significant.

Although NHANES employs a complex multistage sampling framework incorporating sampling weights, strata, and primary sampling units to generate nationally representative estimates, the present analyses were conducted without incorporation of survey‐design weighting variables. Therefore, the reported effect estimates primarily reflect associations within the analytic sample rather than fully weighted national estimates, and the precision of variance estimation may be affected accordingly.

### 2.7. Multivariable Logistic Regression Analysis

The UHR was analyzed both as a continuous variable and by quartiles to improve interpretability of this ratio‐basedindex. Multivariable logistic regression models were constructed to evaluate the association between UHR and CP prevalence.

Model 1 was unadjusted. Model 2 adjusted for demographic covariates, including age, sex, race, and PIR. Model 3 further adjusted for metabolic and clinical covariates, including BMI, WC, TG, LDL, FPG, HbA1C, hypertension, diabetes, SBP, smoking history, and alcohol consumption.

Several covariates incorporated into the fully adjusted models represented related metabolic domains, including BMI, TG, FPG, HbA1C, LDL, and UHR. Although these variables were included to minimize residual metabolic confounding, formal multicollinearity diagnostics were not performed. Therefore, coefficients from multivariable models should be interpreted cautiously, particularly regarding the independence and magnitude of metabolically related predictors.

### 2.8. Nonlinear and Threshold Effect Analyses

Restricted cubic spline analyses were performed to explore potential nonlinear associations between UHR and CP prevalence following multivariable adjustment. Two‐piecewise linear regression models were subsequently applied when nonlinearity was suggested, with the optimal inflection point determined using model likelihood maximization. Threshold effects were evaluated using likelihood ratio tests comparing linear and segmented models.

To improve interpretability of the observed associations, UHR was examined both continuously and by quartiles. Quartile‐based analyses were used to provide clinically interpretable estimates across the observed UHR distribution.

### 2.9. Statistical Software

Data extraction, variable integration, statistical analyses, and figure generation were performed using R software (Version 4.3.2). Logistic regression analyses, spline fitting, and threshold‐effect analyses were conducted using relevant R packages, including “tidyverse,” “rms,” and “segmented.” Tables were generated using the “tableone” and “gtsummary” packages, whereas figures were created using “ggplot2.”

## 3. Results

### 3.1. Demographic and Preliminary Characteristics of Participants

The study included 12,012 participants aged over 20 years from the NHANES database between 1999 and 2004, with 6227 females, representing approximately 51.84% of the study population. Analysis of UHR distribution by gender revealed that higher UHR levels were associated with an increased proportion of males and a decreased proportion of females. Within this cohort, 1748 individuals were classified as having CP, accounting for 14.55% of participants. The majority of the study population were non‐Hispanic Whites, totaling 6169 individuals, or 51.36% of the cohort. Table 1 summarizes the demographic and baseline characteristics stratified by UHR quartiles. Participants with higher UHR values tended to be older (*p* < 0.001), have lower income (*p* < 0.001), higher UA levels (*p* < 0.001), and lower HDL cholesterol (*p* < 0.001). Additionally, significant differences were observed among groups with varying UHR in terms of gender, race (*p* < 0.001), and CP status (*p* = 0.042).

**TABLE 1:** Characteristics of the participants categorized by UHR.

UHR quartile	Q1 (17.00–170.79)	Q2 (170.88–238.79)	Q3 (238.82–327.13)	Q4 (327.14–1995.48)	*p* value
*N*	2997	2999	2994	3003	
Age, years	43.00 (30.00–62.00)	48.00 (33.00–65.00)	49.00 (35.00–65.75)	51.00 (35.00–67.00)	< 0.001
PIR	2.47 (1.28–4.59)	2.23 (1.20–4.13)	2.23 (1.20–4.07)	2.32 (1.22–4.12)	< 0.001
BMI, kg/m^2^	28.00 (24.10–32.50)	28.10 (24.10–32.80)	27.80 (24.00–32.50)	27.80 (24.10–32.40)	0.660
UA, mg/dL	2226.00 (196.30–261.70)	291.50 (255.80–327.10)	333.10 (297.40–374.70)	404.50 (362.80–452.00)	< 0.001
HDL, mmol/L	1.76 (1.53–2.04)	1.42 (1.24–1.60)	1.19 (1.09–1.34)	0.98 (0.85–1.09)	< 0.001
TG, mmol/L	1.34 (1.12–1.34)	1.34 (1.15–1.34)	1.34 (1.12–1.34)	1.34 (1.07–1.34)	0.860
LDL, mmol/L	2.88 (2.85–2.88)	2.88 (2.85–2.88)	2.88 (2.88–2.88)	2.88 (2.87–2.88)	0.665
SBP, mmHg	122.00 (112.00–132.00)	122.00 (112.00–133.00)	122.00 (110.00–132.00)	122.00 (112.00–132.00)	0.811
HBA1C	5.50 (5.20–5.90)	5.50 (5.20–5.90)	5.50 (5.20–5.90)	5.50 (5.20–5.90)	0.851
FPG, mmol/L	6.08 (1.38) 6.10 (5.59–6.10)	6.14 (1.55) 6.10 (5.61–6.10)	6.05 (1.33) 6.10 (5.55–6.10)	6.10 (1.47) 6.10 (5.55–6.10)	0.134
WC, cm	98.30 (87.20–109.30)	997.90 (87.20–109.20)	97.10 (86.70–108.90)	97.10 (86.05–108.90)	0.480
Gender					< 0.001
Male	384 (12.81)	1152 (38.41)	1849 (61.76)	2381 (79.29)	
Female	2613 (87.19)	1847 (61.59)	1145 (38.24)	622 (20.71)	
Race					< 0.001
Mexican	636 (21.22)	695 (23.17)	701 (23.41)	649 (21.61)	
Hispanic	115 (3.84)	143 (4.77)	143 (4.78)	133 (4.43)	
White	1584 (52.85)	1438 (47.95)	1535 (51.27)	1612 (53.68)	
Black	550 (18.35)	624 (20.81)	525 (17.54)	493 (16.42)	
Other Race	112 (3.74)	99 (3.30)	90 (3.01)	116 (3.86)	
Alcohol user, *n* (%)					0.933
Yes	1236 (41.24)	1245 (41.51)	1259 (42.05)	1253 (41.72)	
No	1761 (58.76)	1754 (58.49)	1735 (57.95)	1750 (58.28)	
Smoking status, *n* (%)					0.969
Yes	2199 (73.37)	2194 (73.16)	2191 (73.18)	2212 (73.66)	
No	798 (26.63)	805 (26.84)	803 (26.82)	791 (26.34)	
Hypertension					0.295
Yes	1214 (40.51)	1239 (41.31)	1175 (39.25)	1181 (39.33)	
No	1783 (59.49)	1760 (58.69)	1819 (60.75)	1822 (60.67)	
Diabetes					0.970
Yes	501 (16.72)	496 (16.54)	507 (16.93)	509 (16.95)	
No	2496 (83.28)	2503 (83.46)	2487 (83.07)	2494 (83.05)	
CP					0.042
Yes	403 (13.45)	422 (14.07)	445 (14.86)	478 (15.92)	
No	2594 (86.55)	2577 (85.93)	2549 (85.14)	2525 (84.08)	

### 3.2. Univariate Logistic Analysis Reveals Associations Between Multiple Variables and CP

Univariate logistic regression indicated that age (OR = 1.01, 95% CI: 1.01–1.01, *p* < 0.0001), WC (OR = 1.00, 95% CI: 1.00–1.01, *p* = 0.0018), and BMI (OR = 1.01, 95% CI: 1.00–1.02, *p* = 0.0307) were positively associated with CP, whereas PIR (OR = 0.92, 95% CI: 0.89–0.95, *p* = 0.0009) and HDL (OR = 0.82, 95% CI: 0.72–0.93, *p* = 0.0017) demonstrated negative associations (Table 2). These findings highlight the influence of both demographic and metabolic factors on the prevalence of CP in this cohort.

**TABLE 2:** Weighted univariate logistic analyses between variables and CP (odds ratios, 95% confidence intervals).

Variables	Univariate analysis (crude mode l)	
	OR 95% CI	*p* value
Age	1.01 (1.01, 1.01)	< 0.0001
PIR	0.92 (0.89, 0.95)	< 0.0001
BMI	1.01 (1.00, 1.02)	0.0307
TG	1.02 (0.97, 1.07)	0.3950
UA	1.00 (1.00, 1.00)	0.0054
HDL	0.82 (0.72, 0.93)	0.0017
UHR	1.00 (1.00, 1.00)	0.0016
LDL	1.00 (1.00, 1.00)	0.0054
SBP	1.00 (1.00, 1.00)	0.8336
WC	1.00 (1.00, 1.01)	0.0018

### 3.3. The Relationship Between CP and UHR Quartiles

As presented in Table 3, higher UHR quartiles were linked to a greater prevalence of CP. In the unadjusted model (Model 1), a positive association between UHR quartiles and CP prevalence was observed. After partial adjustment in Model 2, this relationship remained significant. In the fully adjusted model (Model 3), compared with the reference quartile (Q1), the odds ratios for CP in the second (Q2), third (Q3), and fourth (Q4) quartiles were 1.12 (95% CI: 0.96–1.30), 1.27 (95% CI: 1.08–1.48), and 1.44 (95% CI: 1.22–1.70), respectively (*p* < 0.001). When analyzed as a continuous variable, each one‐unit increase in UHR was associated with a 44% higher odds of CP. Given the ratio‐based nature of UHR, quartile‐based comparisons provided a more interpretable summary of the clinical association. The fully adjusted comparison between Q4 and Q1 indicated that participants with higher UHR values across the observed distribution had 44% higher odds of CP.

**TABLE 3:** Multivariable logistic regression analysis of the association between UHR and CP.

	UHR quartiles				*p* for trend
	Q1 (17.00–170.79)	Q2 (170.88–238.79)	Q3 (238.82–327.13)	Q4 (327.14–1995.48)	
	OR (95% CI)	OR (95% CI)	OR (95% CI)	OR (95% CI)	
CP	403 (13.45%)	422 (14.07%)	445 (14.86%)	478 (15.92%)	
Model 1	Reference	1.05 (0.91, 1.22)	1.12 (0.97, 1.30)	1.22 (1.06, 1.41)	< 0.001
Model 2	Reference	1.14 (0.98, 1.33)	1.31 (1.12, 1.53)	1.50 (1.27, 1.76)	< 0.001
Model 3	Reference	1.12 (0.96, 1.30)	1.27 (1.08, 1.48)	1.44 (1.22, 1.70)	< 0.001

*Note:* Values are presented as weighted odds ratios (ORs), 95% confidence interval (95% CI), and *p* value. Model 1 adjusted for none. Model 2 adjusted for gender, age and race. Model 3 adjusted for gender, age, PIR, education level, race, alcohol user, smoking status, BMI, HDL, TG, LDL, SBP, HBA1C, FPG, WC, DM, and hypertension.

**FIGURE 2:**
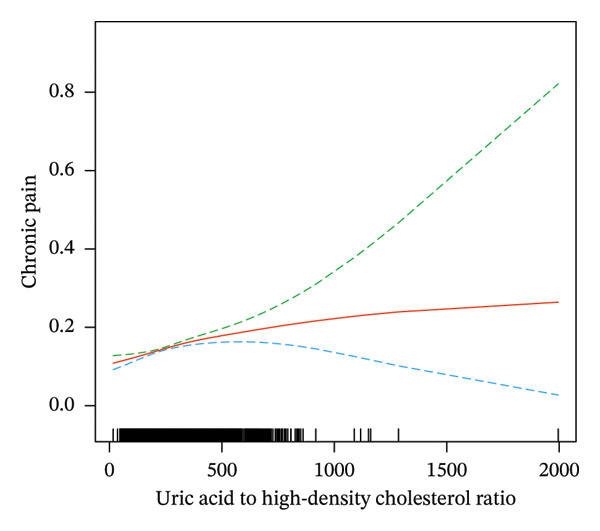
Depiction of the association between the uric acid to HDL cholesterol ratio (UHR) and chronic pain (CP) prevalence.

Smooth‐curve fitting and threshold‐effect analyses were conducted to explore the form of the UHR–CP association. Figure 2 demonstrates an approximately positive linear relationship. Standard linear regression (Model a, Table 4) showed that each one‐unit increase in UHR was associated with an 11% higher odds of CP (*p* = 0.0003). Two‐piecewise linear regression suggested a potential inflection point at a UHR value of 512; For UHR values ≤ 512, each one‐unit increase was associated with an 8% higher odds of CP (OR = 1.08, 95% CI: 1.01–1.11, *p* < 0.0001), whereas no significant association was observed for values > 512 (OR = 1.00, 95% CI: 0.88–1.09, *p* = 0.9606). However, the log‐likelihood ratio test yielded *p* = 0.141, indicating that the segmented model did not significantly improve fit over the simpler linear model. Overall, these analyses support an approximately linear positive association between UHR and CP prevalence.

**TABLE 4:** Threshold and saturation effect analysis of UHR on CP.

Outcomes	CP	
	OR (95% CIs)	*p* value
*Model a*		
(Logistic regression model with UHR as a continuous variable)	1.11 (1.02, 1.13)	0.0003

*Model b*		
(Two‐piecewise logistic regression model)		
Inflection point	512	
≤ Inflection point	1.08 (1.01, 1.11)	< 0.0001
> Inflection point	1.00 (0.88, 1.09)	0.9606
*p* for log‐likelihood ratio test	0.141	

*Note:* The two‐piecewise regression models were adjusted for gender, age, PIR, education level, race, alcohol user, smoking status, BMI, HDL, TG, LDL, SBP, HBA1C, FPG, WC, DM, and hypertension. OR odds ratios, 95% CI 95% confidence interval.

## 4. Discussion

CP is a prevalent symptom with no definitive etiology, yet it causes considerable physical and psychological distress [41, 42]. CP is frequently accompanied by inflammatory dysregulation, and UHR is recognized as a marker reflecting inflammatory status. To date, no studies have specifically explored the relationship between UHR and CP. In our analysis, we observed a significant positive association between UHR and CP prevalence in a large NHANES‐based sample of US adults. This finding aligns with accumulating evidence emphasizing the role of inflammation and metabolic alterations in CP. As a marker of inflammatory and metabolic status, UHR may provide an additional perspective for understanding the biological profile underlying CP.

UA has been implicated in cancer risk. A large Swedish cohort including 493,281 individuals aged ≥ 20 years employed multivariate Cox proportional hazards regression to examine the relationship between gender‐specific UA quartiles and cancer incidence. The study found a positive correlation between serum UA and overall cancer risk, even after adjusting for glucose, TG, and BMI [43]. It has been reported that UA may exert protective effects on cognitive function, particularly in males, and at physiological levels, UA may display anti‐inflammatory properties [44]. Other studies have compared UHR in the context of Hashimoto’s thyroiditis (HT), reporting mean UHR values of 11% ± 4% in HT patients versus 8% ± 2% in controls (*p* < 0.001). UHR positively correlated with TSH (*r* = 0.26, *p* = 0.01) and negatively with FT4 (*r* = −0.22, *p* = 0.04), suggesting that UHR may be associated with conditions such as HT and potentially CP [45].

The observed association between UHR and CP is biologically plausible. UA, a proinflammatory mediator, has been implicated in multiple pain pathways, whereas HDL‐C, with known anti‐inflammatory properties, may mitigate these effects. Our results indicate that as UHR increases, the odds of CP also rise, implying that a proinflammatory state characterized by higher UA and lower HDL‐C may contribute to CP. Exploratory two‐piecewise modeling suggested a potential inflection point near a UHR value of 512; however, the likelihood ratio test did not strongly support a segmented model over a simpler linear model. Therefore, this threshold should be considered hypothesis‐generating, with the overall data supporting an approximately linear positive association. Although the exploratory two‐piecewise model suggested attenuation of the association at very high UHR levels, the segmented model did not significantly outperform the simpler linear model. Therefore, this apparent plateau effect should be interpreted cautiously. One possible explanation is that CP‐related metabolic‐inflammatory signaling may exhibit partial saturation or ceiling effects at higher inflammatory burdens, although this remains speculative and requires mechanistic validation.

Unlike isolated UA or HDL measurements, UHR may simultaneously reflect both increased proinflammatory oxidative burden and impaired anti‐inflammatory buffering capacity, potentially providing a broader representation of the metabolic‐inflammatory milieu relevant to CP. The biological relevance of UHR to CP may extend beyond generalized inflammation to mechanisms involved in pain chronification. Elevated UA is linked to oxidative stress, inflammasome activation, and danger‐associated signaling, which may promote the release of pronociceptive mediators and sustain peripheral neuroimmune activation [46]. Concurrently, reduced HDL‐associated anti‐inflammatory and antioxidant capacity may impair resolution of sterile inflammation and disrupt vascular‐metabolic homeostasis, creating a microenvironment conducive to persistent nociceptive signaling [47]. Continuous peripheral input may further trigger spinal glial activation, cytokine‐driven synaptic plasticity, and maladaptive amplification of nociceptive transmission, central to pain chronification and central sensitization. While the present cross‐sectional study cannot confirm direct mechanistic participation of UHR in these pathways, the observed association is consistent with the concept that metabolic‐inflammatory imbalance may interact with neuroimmune signaling and oxidative stress to maintain CP [48].

Strengths of this study include the large sample size and rigorous adjustment for demographic, clinical, and lifestyle confounders. Nevertheless, the operational definition of CP based on NHANES questionnaires necessitates caution. It likely captures a heterogeneous population with diverse pain mechanisms, anatomical distributions, and underlying causes, potentially attenuating phenotype‐specific associations between UHR and CP. Reliance on self‐reported pain may introduce misclassification, particularly in individuals with fluctuating symptoms or difficulty recalling pain duration. Additionally, although NHANES utilizes a complex multistage sampling design, the complete‐case analysis did not incorporate sampling weights, strata, or primary sampling units, so observed associations may not fully represent national estimates. Covariates in the fully adjusted models were metabolically interrelated, and UHR itself is a composite index; formal collinearity diagnostics were not performed, so multicollinearity cannot be excluded, which may affect coefficient stability. Future studies should apply diagnostic measures such as variance inflation factors or tolerance statistics. The cross‐sectional design also precludes causal inference, highlighting the need for longitudinal studies with more refined pain phenotyping.

Chronic pain is a multidimensional condition influenced not only by duration but also by severity, body distribution, functional impairment, affective burden, and etiology. The NHANES‐based definition cannot distinguish localized musculoskeletal, neuropathic‐like, visceral, or mixed pain types, nor quantify disability or interference with daily life. Consequently, these findings reflect a broad CP construct rather than a specific clinical phenotype. Continuous‐model coefficients summarize overall positive trends, while quartile‐based comparisons offer more interpretable effect sizes, given that UHR lacks established CP‐specific cutoffs.

A substantial number of participants were excluded due to missing covariate information, which may have introduced selection bias and reduced generalizability. Complete‐case analysis assumes excluded observations are similar to included ones; if missingness correlated with metabolic status, pain burden, or clinical features, bias may exist. Future analyses using multiple imputation or sensitivity approaches are warranted. Some potentially relevant covariates, including physical activity, depressive symptoms, sleep disturbance, renal function, and analgesic use, were not incorporated, so residual confounding cannot be ruled out.

The implications of these findings are primarily scientific rather than immediately clinical. UHR may serve as an accessible metabolic indicator associated with CP, supporting further investigation into metabolic and inflammatory pathways contributing to pain. However, no diagnostic model was developed or validated, and discrimination or calibration performance was not assessed, so the results do not support immediate clinical application or AI‐driven implementation [49, 50].

## 5. Conclusion

The present study identified a significant positive association between elevated UHR and CP prevalence in US adults. These findings support a link between metabolic‐inflammatory status and CP, while suggesting that composite metabolic‐inflammatory indices may have relevance in pain‐related epidemiologic research. Given the cross‐sectional design and the heterogeneous nature of CP phenotypes captured in NHANES, causal interpretation should be avoided, and residual confounding related to systemic inflammation or metabolic dysfunction cannot be fully excluded. Further longitudinal, mechanistic, and comparative studies incorporating established inflammatory biomarkers will be necessary to clarify the biological specificity and potential clinical relevance of UHR in CP.

## Author Contributions

Zifeng Xu and Jianwei Wang oversaw the study’s conception and design. Yanjun Liu analyzed and interpreted the gathered data. Yanjun Liu authored the manuscript outlining their research findings.

## Funding

No funding was received for this manuscript.

## Disclosure

All authors significantly contributed to the paper and collectively approved the final version submitted for publication.

## Ethics Statement

The investigation was approved by the National Center for Health Statistics (NCHS) Research Ethics Review Board, and all participants provided written informed consent.

## Consent

The authors have nothing to report.

## Conflicts of Interest

The authors declare no conflicts of interest.

## Supporting Information

Additional supporting information can be found online in the Supporting Information section.

## Supporting information


**Supporting Information** Supporting Table 1 (UHR_CP): This file provides the raw dataset used in the analysis, including participants’ demographic information, laboratory measurements (uric acid, HDL cholesterol, and computed UHR), chronic pain status, and relevant covariates such as BMI, blood pressure, diabetes status, and smoking history. Data were obtained from NHANES cycles spanning 1999–2004 and support the findings reported in this manuscript.

## Data Availability

The data used in this study are publicly available from the National Health and Nutrition Examination Survey (NHANES) at: https://www.cdc.gov/nchs/nhanes/index.htm.
